# Analysis of Naturally Occurring Phenolic Compounds in Aromatic Plants by RP-HPLC Coupled to Diode Array Detector (DAD) and GC-MS after Silylation

**DOI:** 10.3390/foods2010090

**Published:** 2013-03-13

**Authors:** Charalampos Proestos, Michael Komaitis

**Affiliations:** 1 Laboratory of Food Chemistry, Department of Chemistry, National and Kapodistrian University of Athens, 15771, Athens, Greece; 2 Laboratory of Food Chemistry and Analysis, Department of Food Science and Technology, Agricultural University of Athens, 11855, Athens, Greece; E-Mail: achem@aua.gr

**Keywords:** aromatic plants, phenolic compounds, RP-HPLC, GC-MS, silylation

## Abstract

The following aromatic plants of Greek origin, *Origanum dictamnus* (dictamus), *Eucalyptus globulus* (eucalyptus), *Origanum vulgare* L. (oregano), *Mellisa officinalis* L. (balm mint) and *Sideritis cretica* (mountain tea), were examined for the content of phenolic substances. Reversed phase HPLC coupled to diode array detector (DAD) was used for the analysis of the plant extracts. The gas chromatography-mass spectrometry method (GC-MS) was also used for identification of phenolic compounds after silylation. The most abundant phenolic acids were: gallic acid (1.5–2.6 mg/100 g dry sample), ferulic acid (0.34–6.9 mg/100 g dry sample) and caffeic acid (1.0–13.8 mg/100 g dry sample). (+)-Catechin and (−)-epicatechin were the main flavonoids identified in oregano and mountain tea. Quercetin was detected only in eucalyptus and mountain tea.

## 1. Introduction

Phenolic compounds are almost ubiquitous in plant foods (cereals, vegetables, fruits, legumes, nuts, *etc.*) and beverages (wine, beer, tea, cocoa, cider, *etc.*) [1]. Their levels vary greatly even between cultivars of the same species. For example, the formation of flavone and flavonol glycosides greatly depends on light; therefore, the highest concentrations of these compounds are found generally in leaves and outer parts of plants, with only trace amounts in the subterranean parts of plants [2]. The presence of polyphenols in plant foods is largely influenced by genetic factors and environmental conditions. Other factors, such as germination, degree of ripeness, variety, processing, and storage, also influence the content of plant phenolics [3,4]. 

Until recently, most of the nutritional interest in polyphenolic compounds was in the deleterious effects caused by the ability of certain polyphenols to bind and precipitate macromolecules, such as dietary protein, carbohydrate and digestive enzymes, thereby reducing food digestibility. Recent interest, however, in food phenolics has increased greatly because of the antioxidant and free radical-scavenging abilities associated with some phenolics and their potential effects on human health [5]. Phenolic compounds can be found free, although their corresponding methyl and ethyl esters and glycosides occur very commonly in free and/or bound forms [5]. Phenylpropanoid derivatives (C_6_C_3_) also are an important group of low-molecular-weight phenolics. Chromones are less known compared with coumarins, with the latter occurring naturally as glycosides (e.g., umbelliferone, aesculetin, scopoletin). The most important phenylpropanoids are the hydroxycinnamic acids (*p*-coumaric, caffeic, ferulic, sinapic) and derivatives. Cinnamyl alcohols (coniferyl alcohol or guaiacyl, sinapyl alcohol or syringyl, and *p*-coumaryl alcohol *p*-hydroxyphenyl) form the basic constituent of lignins, and thus represent one of the major groups of plant phenolics. Phenylpropanoids and more simple phenols (benzoic acid and benzaldehyde derivatives) are usually covalently linked to cell wall polysaccharides (predominantly ester-linked to arabinose units of hemicellulose) or to the so-called core lignin [6,7]. Flavonoids represent the most common and widely distributed group of plant phenolics. Their common structure is that of diphenylpropanes (C_6_C_3_C_6_) and consists of two aromatic rings linked through three carbons that usually form an oxygenated heterocycle. Biogenetically, the A ring usually comes from a molecule of resorcinol or phloroglucinol synthesized in the acetate pathway, whereas ring B is derived from the shikimate pathway [5]. Flavonoids occasionally occur in plants as aglycones, although they are most commonly found as glycoside derivatives. Among the flavonoids, flavones (e.g., apigenin, luteolin, diosmetin), flavonols (e.g., quercetin, myricetin, kaempferol), and their glycosides are the most common compounds. They are widespread in the plant kingdom, with the exception of algae and fungi. Flavonols occur as *O*-glycosides, but flavone *O*-glycosides and *C*-glycosides are very common [2], with the latter characterized for possessing a carbon-carbon linkage between the anomeric carbon of a sugar molecule and the C-6 or C-8 carbon of the flavone nucleus. Unlike *O*-glycosides, sugars in *C*-glycosides are not cleaved by acid hydrolysis. Flavanones (e.g., naringenin, hesperidin) also can occur as *O*- or *C*-glycosides and are especially abundant in citrus foods and prunes. The variability of this group of flavonoids is noteworthy, with about 380 flavonol glycosides and 200 different quercetin and kaempherol glycosides described to date [8]. Dictamnus, is an aromatic plant found in Crete and it was used from ancient times against stomach pains and as an effective painkiller and wound healing agent. Eucalyptus is one of the most known aromatic plants in the world trade market. It grows in Australia and in Mediterranean countries. Oregano is very often used in Greek cuisine as seasoning due to its strong flavor. A green tea, such as sideritis, as well as black tea have been examined by many researchers for their content in polyphenols. In plant materials is difficult to determine individual flavonoid glycosides. Therefore, the glycosides are hydrolyzed and the resulting aglycones are identified and quantified [9]. A number of analytical methods have been proposed for the separation and determination of phenolic compounds mainly based on a high performance liquid chromatography (HPLC) technique with UV spectrophotometry because derivatization is not required prior to analysis [10,11,12]. However, compared to mass spectrometry (MS), the UV-Vis spectrum does not supply sufficient identifying power [13,14]. Hence, gas chromatography coupled with mass spectometry (GC-MS) can provide more accurate results. Both methods were used for the analysis of plant extracts. Analysis of the non volatile and thermolabile phenolic compounds by GC-MS presupposes their conversion into volatile and thermotolerant ones by chemical derivatization [15].

## 2. Materials and Methods

### 2.1. Standards

Gallic acid, gentisic acid, *p*-coumaric acid, vanillic acid, ferulic acid, (+)-catechin, quercetin, apigenin, naringenin, eriodictyol were purchased from Sigma-Aldrich (Steinheim, Germany). Luteolin was from Roth (Karlsruhe, Germany). Caffeic acid was from Merck (Darmstadt, Germany). Rutin was from Alexis biochemicals (Lausen, Switzerland). Hydroxytyrosol, tyrosol, *p*-hydroxybenzoic acid and BHT (butylated hydroxytoluene) were a kind donation from the National Agricultural Research Foundation (N.AG.RE.F, Greece). All standards were prepared as stock solutions in methanol. Working standards were made by diluting stock solutions in 62.5% aqueous methanol containing BHT 1 g/L, and 6 M HCL to yield concentrations ranging between 0.5–25 mg/L. Stock working solutions of the standards were stored in darkness at −18 °C.

### 2.2. Solvents and Reagents

All solvents and reagents from various suppliers were of the highest purity needed for each application. Silylation reagents, BSTFA (*N*,*O*-bis(trimethylsilyl)trifluoroacetamide), TMCS (trimethylchlorosilane), and HMDS (hexamethyldisilazane) were purchased from Merck (Darmstadt, Germany), respectively. 5% DMDCS (dimethyldichlorosilane) in toluene (used for deactivating glassware surfaces) was obtained from Sigma-Aldrich (Steinheim, Germany).

### 2.3. Samples

Samples of *Origanum dictamnus* (dictamnus), *Eucalyptus globulus* (eucalyptus), *Origanum vulgare* L. (oregano), *Mellisa officinalis* L. (balm mint) and *Sideritis cretica* (mountain tea) were obtained from local stores. Leaves were dried at 25 °C in darkness and analyzed after grinding in a household blender. All samples were analyzed within 3 months of collection.

### 2.4. Sample Preparation and Derivatization

The extraction method used for dried samples had as follows: 40 mL of 62.5% aqueous methanol containing BHT (1 g/L) was added to 0.5 g of dried sample. Then 10 mL of 6 M HCL were added. The mixture was stirred carefully. In each sample, nitrogen was bubbled for *ca.* 40–60 s. The extraction mixture was then sonicated for 15 min and refluxed in a water bath at 90 °C for 2 h. The mixture was then: (a) filtered and made up to 100 mL with methanol [10,11], furthermore filtered quickly through a 0.45 μm membrane filter membrane filter (Millex-HV, Millipore Corporation, Billerica, MA, USA) and injected to HPLC or (b) extracted with 30 mL (3 × 10 mL) ethyl acetate. The organic layer was collected and reduced to 10 mL by rotary evaporation (37 °C) and centrifuged for 10 min. Anhydrous Na_2_SO_4_ was then added to remove moisture. Then, 100 μL of the organic layer were derivatizized after evaporation of the solvent under nitrogen stream. For the silylation procedure a mixture of TMCS (100 μL) and BSTFA (200 μL) were added and vortexed in screw cap glass tubes (priory deactivated with 5% DMDCS in toluene, and rinsed two times with toluene and three times with methanol), and consecutively placed in a water bath at 80 °C for 45 min. From the silylated mixture 1 μL was directly analyzed by GC-MS. To prevent enzymic oxidation, extraction of the polyphenols from plants with boiling alcohol is essential and should be adopted routinely [9]. For the same reason all this work was carried out in dark and under nitrogen atmosphere. 

### 2.5. HPLC Analysis

The analytical HPLC system employed consisted of a JASCO high performance liquid chromatograph coupled with a diode array detector (MD-910 JASCO, Tokyo, Japan). The analytical data were evaluated using a JASCO data processing system (DP-L910/V). The separation was achieved on a Waters Spherisorb^®^ 5 μm ODS2 4.6 × 250 mm column (Milford, MA, USA) at ambient temperature. The mobile phase consisted of water with 1% glacial acetic acid (solvent A), water with 6% glacial acetic acid (solvent B), and water-acetonitrile (65:30 v/v) with 5% glacial acetic acid (solvent C). The gradient used was similar to that used for the determination of phenolics in wine [16] with some modifications: 100% A, 0–10 min; 100% B, 10–30 min; 90% B/10% C, 30–50 min; 80% B/20% C, 50–60 min; 70% B/30% C, 60–70 min; 100% C, 70–105 min; 100% A, 105–110 min; post time 10 min before next injection. The flow rate was 0.5 mL/min and the injection volume was 20 μL. The monitoring wavelength was 280 nm for the phenolic acids and 320 and 370 nm (flavones, flavonoles). The identification of each compound was based on a combination of retention time and spectral matching.

### 2.6. GC-MS Analysis

The silylated samples were injected into a GC-MS system consisted of a Fisons GC 8000 Series (ThermoQuest, Milan, Italy), model 8060 gas chromatograph coupled with a Fisons MD 800 mass spectrometer in the EI (Electron Impact) mode with the electron energy set at 70 eV and the mass range at *m/z* 25–700. A capillary column Low-bleed CP-Sil 8 CB-MS (30 m × 0.32 mm, i.d.), of 0.25 μm film thickness of coated material was used. The injector was set at 280 °C and the detector at 290 °C. GC was performed in the splitless mode with 1 min splitless-time. The temperature program was as follows: from 70 °C to 135 °C with 2 °C/min, hold for 10 min; from 135 °C to 220 °C with 4 °C/min, hold for 10 min; from 220 °C to 270 °C with 3.5 °C/min and then hold for 20 min. A post run of 10 min at 70 °C was sufficient for the next injection. The flow rate of carrier gas (helium) was maintained at 1.9 mL/min. Identification of compounds was obtained by comparing the retention times with those of authentic compounds and the spectral data obtained from the Wiley and NIST libraries. Each determination was carried out in duplicate.

## 3. Results and Discussion

### 3.1. HPLC Analysis

**Figure 1:**
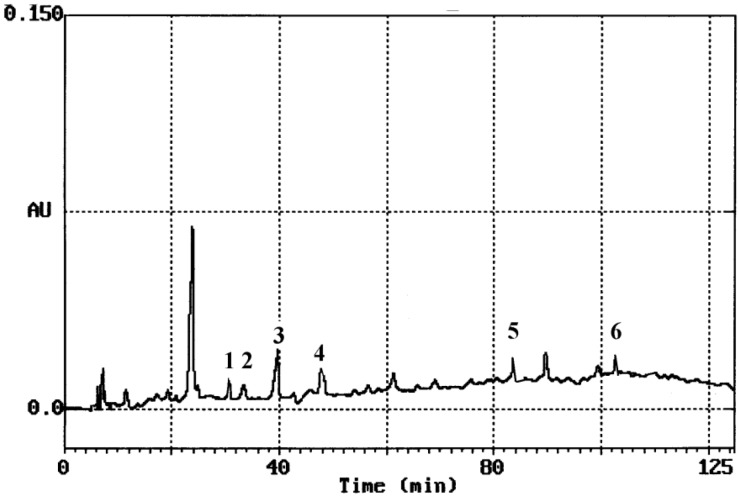
Typical HPLC chromatograph of *M. officinalis* L. where: 1, gentistic acid; 2, *p*-hydroxybenzoic acid; 3, (+)-catechin; 4, caffeic acid; 5, ferulic acid; 6, eriodictyol.

Hydrolysis of glycosides or esters was necessary, so as to determine phenolic content by HPLC, since a considerable fraction is in bound form [8]. Extraction was performed with a mixture of 62.5% aqueous methanol. Methanol has a protective role. It can prevent phenolic compounds from being oxidized by enzymes, such as phenoloxidases [9]. Columns employed to separate phenolics are almost exclusively reversed-phase. The identification of each compound was based on a combination of retention time and spectral matching, since polyphenols absorb in the ultraviolet (UV) region. According to the literature, the most of the benzoic acid derivatives show absorption maximum at 246–262 nm with a shoulder at 290–315 nm, except gallic acid that shows a maximum at 271 nm [8]. Two absorption bands are characteristic of flavonoids. Band II with a maximum in the 240–285 nm range, is believed to arise from the A-ring. Band I, with a maximum in the 300–550 nm range is attributed to the B-ring [12]. After extraction and acid hydrolysis the content of phenolic substances was determined by HPLC quantitative analysis. A typical HPLC chromatogram of *M. officinalis* L. (balm mint) is presented in Figure 1.

The amounts of phenolic compounds detected in the samples are presented in Table 1. Results are expressed in mg/100 g dry sample. The most abundant phenolic acids were: gallic acid (1.5–2.6 mg/100 g dry sample), ferulic acid (0.34–6.9 mg/100 g dry sample) and caffeic acid (1.0–13.8 mg/100 g dry sample). (+)-Catechin and (−)-epicatechin were the main flavonoids identified in oregano and mountain tea. Quercetin was detected only in eucalyptus and mountain tea. Similar results within other aromatic plants have been previously presented [17].

**Table 1:** Content of phenolic compounds in the examined plant extracts (in 62.5% aqueous methanol)

Content, mg/100 g dry sample ^a^								
Plant	Gallic acid	Gentisic acid	Caffeic acid	*p*-Coumaric acid	Vanillic acid	Syringic acid	Ferulic acid	*p*-Hydroxybenzoic acid
*O. dictamnus*	ND	ND	ND	13.9 ± 0.04	ND	ND	0.34 ± 0.01	ND
*E. globulus*	1.5 ± 0.03	ND	ND	6.6 ± 0.02	ND	ND	ND	ND
*O. vulgare* L.	ND	ND	1.0 ± 0.02	ND	1.0 ± 0.02	ND	3.2 ± 0.03	ND
*M. officinalis* L.	ND	2.1 ± 0.03	13.8 ± 0.1	ND	ND	ND	4.8 ± 0.03	2.3 ± 0.01
*S. cretica*	2.6 ± 0.02	ND	ND	4.1 ± 0.02	ND	1.1 ± 0.02	6.95	ND
**Plant**	**Quercetin**	**Apigenin**	**Luteolin**	**Naringenin**	**Eriodictyol**	**Rutin**	**(+)-Catechin hydrated**	**(−)-Epicatechin**
*O. dictamnus*	ND	ND	ND	ND	ND	ND	0.5 ± 0.01	ND
*E. globulus*	2.5 ± 0.02	ND	ND	ND	ND	1.8 ± 0.03	ND	ND
*O.vulgare* L.	ND	ND	ND	ND	0.7 ± 0.03	1.0 ± 0.02	17.7 ± 0.1	1.8 ± 0.05
*M. officinalis* L.	ND	ND	ND	ND	1.1 ± 0.05	ND	21.0 ± 0.15	ND
*S. cretica*	1.6 ± 0.04	ND	9.1 ± 0.02	ND	ND	ND	22.1 ± 0.17	6.9 ± 0.06

^a^ Each value is the mean (mg/100 g dry sample) of three replications; ±, Standard deviation; ND, Not detected.

### 3.2. GC-MS Analysis

Silylation is an ideal procedure for the GC analysis of non-volatile and thermolabile compounds. Compared to their parent compounds, trimethylsilyl TMS derivatives are more volatile, less polar and more thermotolerant. In silylation, an active hydrogen in –OH, –COOH, =NH, –NH2 or –SH is replaced by a trimethylsilyl group. Silylation is a nucleophilic substitution reaction. It is viewed as a nucleophilic attack upon the silicon atom of the silyl donor, producing a bimolecular transition state. The silyl compound leaving group must be of low basicity and able to stabilize a negative charge in the transition state [18,19]. BSTFA has been used for the derivatization of phenolic constituents in wines [16], and in white juices and wines from Spain [20]. In our study the temperature and reaction time used was sufficient for the silylation of phenolic compounds.

**Figure 2:**
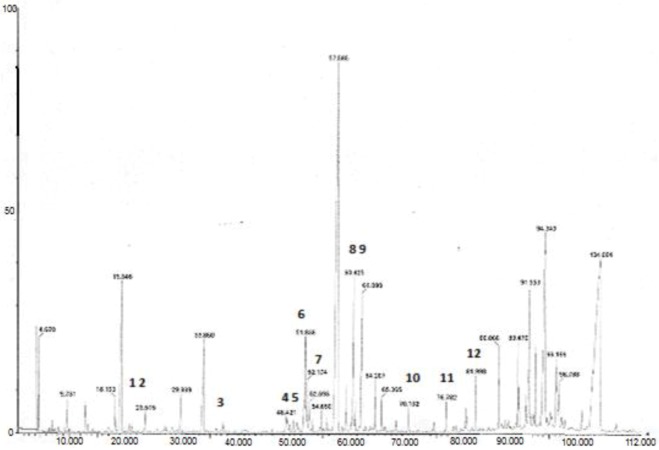
Typical GC/MS chromatograph of *O. vulgare* L. where: 1, cinnamic acid; 2, homogentisic acid; 3, *p*-hydroxybenzoic acid; 4, vanillic acid; 5, gentisic acid; 6, *p*-coumaric acid; 7, protocatechuic acid; 8, *p*-coumaric acid; 9, hydroxycaffeic acid; 10, gallic acid; 11, caffeic acid; 12, 3-nitro-phthalic acid.

The GC oven temperature program, as well as the injector and detector temperatures, was based on previous experience with the analysis of marker compounds in *Ginkgo biloba* L. extract [19]. Prior to employing GC-MS for the determination of phenolic compounds in plant extracts a standard mixture of all substances was tested, after derivatization. Molecular weights (MW) and important ions present in the mass spectra of silylated phenolic compounds in the examined plant extracts are shown in Table 2. Data obtained showed excellent resolution between all compounds of interest. Compounds such as *p*-hydroxyphenylacetic acid, protocatechuic acid and cinnamic acid were detected in *Origanum dictamnus* and *M. officinalis* L. by GC-MS but not by HPLC. A typical GC/MS chromatogram of *O. vulgare* L. is presentd in Figure 2. The molecular ion [M]^+^ for all of the TMS derivatives is a prominent peak in the mass spectrum. Generation of the [M-15] fragment (loss of a methyl group via a-cleavage) and the [M-59] fragment (subsequent loss of CO_2_ after rearrangement) are well established cleavage patterns for TMS esters. Loss of TMSO, [M-89], is also a fragmentation pathway common for derivatized carboxylic acids, because the acylium cation formed is a stable species. Derivatives possessing a methoxy group on the phenyl ring, such as vanillic, ferulic and syringic acid, produce the [M-30] fragment, which represents the loss of a molecule of formaldehyde. For gallic, caffeic and protocatechuic acids, the major fragmentation route generates a predominant [M-177] peak. However, there are no reports describing the origin of this fragment for phenolic acids [21]. The base peak in all mass spectra, except from that of protocatechuic, was the fragment with *m/z* 73, representing the TMS group. Also the presence of the fragment *m/z* 147 that is for the structure [(CH_3_)_2_Si=O–Si(CH_3_)_3_]^+^, means that two or more TMS groups are present in the molecule [22]. The characteristic fragment *m/z* 368 present in catechin and epicatechin represents the retro-Diels Alder cleavage of the B ring and it is characteristic for flavonoids [23]. A possible fragmentation mechanism for silylated tyrosol and hydroxytyrosol has been proposed by other authors [24].

**Table 2:** Molecular weights (MW) and important ions present in the mass spectra of silylated phenolic compounds ^a^ in the examined plant extracts by GC-MS.

Phenolic compound	MW (Silylated compounds)	Molecular ion [M]^+^	Identified ions (*m/z*)
*p*-Hydroxybenzoic acid	282	282	193 (79), 223 (82), 267 (100), 282 (21)
Vanillic acid	312	312	149 (100), 165 (52), 223 (66), 312 (20)
Gentisic acid	370	370	147 (90), 223 (24), 267 (36), 281 (21), 355 (100), 370 (19)
Gallic acid	458	458	147 (94), 179 (48), 281 (100), 458 (32)
*p*-Coumaric acid	308	308	219 (100), 249 (43), 293 (72), 308 (56)
Ferulic acid	338	338	219 (22), 249 (77), 293 (16), 279 (13), 308 (56), 323 (60), 338 (100)
Caffeic acid	396	396	179 (13), 191 (27), 219 (100), 396 (28)
Quercetin	647	647	487 (30), 559 (12), 575 (100), 647 (24)
(+)-catechin	650	650	179 (23), 267 (11), 355 (33), 368 (100), 650 (<1)
Hydroxytyrosol	370	370	179 (33), 193 (43), 267 (100), 370 (23)
Syringic acid	342	342	297 (71), 312 (70), 327 (100), 342 (69)
(−)-Epicatechin	650	650	147 (19), 267 (15), 355 (30), 368 (100), 649 (3)
*p*-Hydroxyphenylacetic acid	296	296	149 (59), 164 (71), 179 (100), 296 (16)
Protocatechuic acid	370	370	193 (100), 223 (24), 267 (12), 370 (15)
Cinnamic acid	220	220	103 (75), 131 (100), 161 (52), 205 (96), 220 (21)

^a^ Identified as TMS derivatives.

## 4. Conclusions

The presence of phenolic compounds, usually called polyphenols, in aromatic plants was proved by employing two different chemical analytical methods. Reversed phase high performance liquid chromatography coupled with a UV-vis multiwavelength detector enables the collection of on-line spectra and simultaneous quantification by several wavelengths. This experiment proved that silyl derivatization offers a very good alternative for the identification of phenolic compounds. However, it should be stressed that more research is needed towards the identification of silyl derivatives. It is believed that this procedure will solve many problems regarding not only the determination of phenolics but also their fate in foodstuffs. 
